# Neuron collinearity differentiates human hippocampal subregions: a validated deep learning approach

**DOI:** 10.1093/braincomms/fcae296

**Published:** 2024-09-03

**Authors:** Jan Oltmer, Emily M Williams, Stefan Groha, Emma W Rosenblum, Jessica Roy, Josue Llamas-Rodriguez, Valentina Perosa, Samantha N Champion, Matthew P Frosch, Jean C Augustinack

**Affiliations:** Department of Radiology, Athinoula A. Martinos Center, Massachusetts General Hospital, Charlestown, MA 02129, USA; Harvard Medical School, Boston, MA 02115, USA; Department of Digital Health and Innovation, Vivantes Netzwerk für Gesundheit GmbH, 13407 Berlin, Germany; Department of Radiology, Athinoula A. Martinos Center, Massachusetts General Hospital, Charlestown, MA 02129, USA; Harvard Medical School, Boston, MA 02115, USA; Division of Population Sciences, Department of Medical Oncology, Dana-Farber Cancer Institute, Boston, MA 02215, USA; Department of Radiology, Athinoula A. Martinos Center, Massachusetts General Hospital, Charlestown, MA 02129, USA; Department of Radiology, Athinoula A. Martinos Center, Massachusetts General Hospital, Charlestown, MA 02129, USA; Department of Radiology, Athinoula A. Martinos Center, Massachusetts General Hospital, Charlestown, MA 02129, USA; J. Philip Kistler Stroke Research Center, Department of Neurology, Massachusetts General Hospital, Harvard Medical School, Boston, MA 02114, USA; C.S. Kubik Laboratory for Neuropathology, Massachusetts General Hospital, Boston, MA 02129, USA; C.S. Kubik Laboratory for Neuropathology, Massachusetts General Hospital, Boston, MA 02129, USA; Department of Radiology, Athinoula A. Martinos Center, Massachusetts General Hospital, Charlestown, MA 02129, USA; Harvard Medical School, Boston, MA 02115, USA

**Keywords:** neuron estimation, pyramidal cell, Cellpose, subregions, algorithm

## Abstract

The hippocampus is heterogeneous in its architecture. It contributes to cognitive processes such as memory and spatial navigation and is susceptible to neurodegenerative disease. Cytoarchitectural features such as neuron size and neuronal collinearity have been used to parcellate the hippocampal subregions. Moreover, pyramidal neuron orientation (orientation of one individual neuron) and collinearity (how neurons align) have been investigated as a measure of disease in schizophrenia. However, a comprehensive quantitative study of pyramidal neuron orientation and collinearity within the hippocampal subregions has not yet been conducted. In this study, we present a high-throughput deep learning approach for the automated extraction of pyramidal neuron orientation in the hippocampal subregions. Based on the pretrained Cellpose algorithm for cellular segmentation, we measured 479 873 pyramidal neurons in 168 hippocampal partitions. We corrected the neuron orientation estimates to account for the curvature of the hippocampus and generated collinearity measures suitable for inter- and intra-individual comparisons. Our deep learning results were validated with manual orientation assessment. This study presents a quantitative metric of pyramidal neuron collinearity within the hippocampus. It reveals significant differences among the individual hippocampal subregions (*P*  *<* 0.001), with cornu ammonis 3 being the most collinear, followed by cornu ammonis 2, cornu ammonis 1, the medial/uncal subregions and subiculum. Our data establishes pyramidal neuron collinearity as a quantitative parameter for hippocampal subregion segmentation, including the differentiation of cornu ammonis 2 and cornu ammonis 3. This novel deep learning approach could facilitate large-scale multicentric analyses in subregion parcellation and lays groundwork for the investigation of mental illnesses at the cellular level.

## Teaser

Oltmer *et al*. present a deep learning pipeline for measuring pyramidal neuron collinearity (orientation) in the hippocampus. The approach corrects for the complex hippocampal morphometry. It was validated with manual measures, reveals significant differences among the hippocampal subregions and potentially aids with the evaluation of disease on the cellular level.

## Graphical Abstract

**Graphical Abstract:**
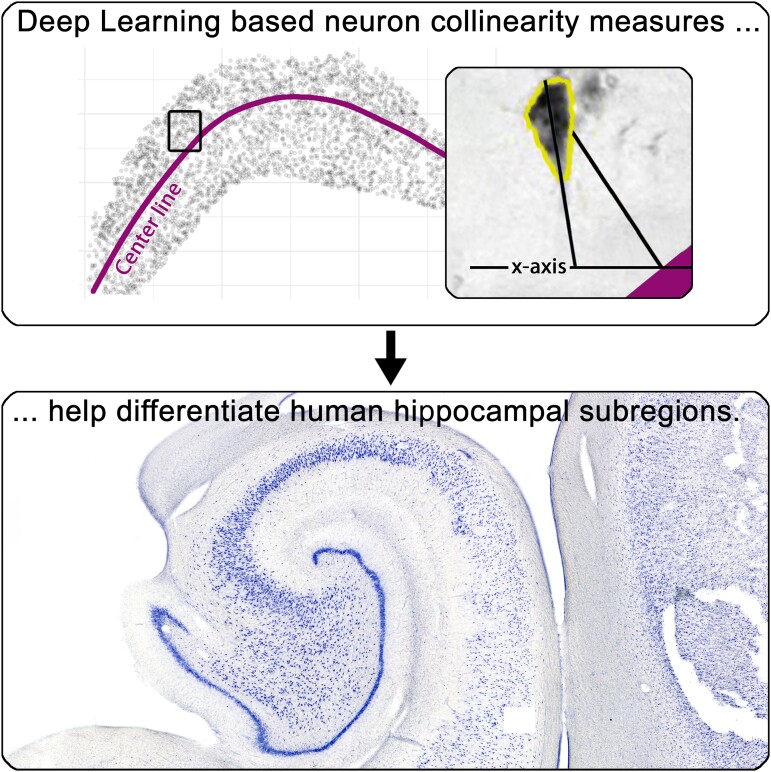

## Introduction

The hippocampus plays a crucial role in cognitive processes such as spatial navigation, memory and learning.^1-4^ However, the hippocampus is not a homogeneous entity. Its morphometric structure is complex and can be segmented into several subregions that differ in functional specialization,^5,6^ susceptibility to Alzheimer’s disease^7-9^ and cytoarchitecture.^10-15^

A major criterion for differentiating hippocampal subregions is pyramidal neuron size.^10-13,16^ While neuron size helps to effectively distinguish certain hippocampal subregions such as cornu ammonis 1 (CA1) and cornu ammonis 2 (CA2), it fails to demarcate a border between CA2 and cornu ammonis 3 (CA3).^11,13^ This highlights the necessity of a multi-parameter approach for hippocampal subsegmentation, taking into account additional pyramidal neuron facets such as neuronal orientation and collinearity.^13^ While the term neuron orientation refers to the orientation of one individual neuron, the term collinearity refers to the same trait in the plural form as a population of neurons (many neurons collectively oriented in direction).

It has been suggested that the hippocampus is a key element in the pathophysiology and neuropathology of the mental illness schizophrenia.^17,18^ Post-mortem analyses from patients suffering from schizophrenia revealed a loss of interneurons in the hippocampus^19^ as well as deviations in hippocampal pyramidal neuron orientation.^20-22^ These findings suggest that subtle deviations from the norm may play a role in mental illness. The disease state may not be as obvious as the changes observed in neurodegenerative processes such as Parkinson’s or Alzheimer’s disease. If it is more subtle than the human eye easily detects, then the ability to evaluate neuronal orientation in multiple populations utilizing a high-throughput approach is attractive to understanding hippocampal variability in health and disease, especially mental health. Notably, though, high-throughput methods such as deep learning require validation from gold standard approaches in microscopy and expert evaluation.

Our recent study presented a deep learning pipeline for segmenting pyramidal neurons from the background in Nissl-stained hippocampal sections utilizing the Cellpose algorithm.^23^ We implemented a custom-automated filtering method to remove false-positive segmentations. This enabled us to generate automated pyramidal neuron estimates and correlate them with stereology counts in the hippocampal subregions, validating the automated deep learning approach.^24^ This study builds on our previous study and presents a deep learning method to assess hippocampal neuron orientation in cognitive controls. In addition, we establish a computational procedure to account for the curvature of the hippocampus in automated orientation estimates. This procedure corrects for the complex morphometry of the hippocampus, which enables us to compute collinearity measures among neurons within the hippocampal subregions and collectively report deviations in neuron orientation within the hippocampal ribbon. We demonstrate orientation data that validates automated neuron collinearity as a novel quantitative metric for differentiating hippocampal subregions, especially CA2 and CA3.

## Materials and methods

### Tissue samples

Seven hemispheres were obtained from the Neuropathology Autopsy Service at Massachusetts General Hospital (age 68.33 ± 14.39, mean ± SD; four males, two females, one sex not available; post-mortem interval < 24 h).^24^ Tissue was gathered from autopsies in which the terms of content allowed to be used for research and all procedures regarding collection of tissue were approved by the Institutional Review Board for Mass General Brigham. Comorbidities were characterized by full neuropathologic autopsy assessment including the presence of neurodegenerative diseases by the Neuropathology Service of the Massachusetts General Hospital; assessment was made including definitive assessment for Alzheimer’s disease neuropathologic changes according to the current guidelines.^25^ Cases were classified as cognitively healthy based on medical history, and cases with psychiatric, neurological or infectious diseases were excluded. The hemispheres were fixed in 10% formalin and underwent gross tissue inspection. Staining with Luxol fast blue and haematoxylin and eosin was performed to rule out vascular disease and stroke. Immunohistochemistry for phosphorylated tau was conducted, and each case was staged for Braak and Braak (BB) by M.P.F. and J.C.A. (one normal control, three BBI and three BBII).^9,26,27^ Table 1 provides a summary of our cases. Supplementary Table 1 lists the reagents used in our experiments.

**Table 1:** Basic demographic information for cases used in study

Case #	Age	Hemisphere	Sex	PMI	Braak and Braak	MTL amyloid burden	Cause of death	Clinical diagnosis
1	43	LH	F	24 h	NC	No	Ischaemic renal injury	Cognitive control
2	84	LH	F	24 h	BBII	No	Pneumonia	Cognitive control
3	59	LH	M	20 h	BBI	No	Liver failure	Cognitive control
4	79	LH	M	15 h	BBI	High	Surgery complication	Cognitive control
5	75	LH	M	24 h	BBII	Moderate	Vascular disease	Cognitive control
6	N/A	LH	N/A	24 h	BBII	No	N/A	Cognitive control
7	68	RH	M	24 h	BBI	No	Acute cardiac death	Cognitive control

### Histology processing

The histology processing followed previously established protocols.^28^ Tissue blocks were cryoprotected in 20% glycerol and 2% dimethyl sulphoxide and sectioned coronally at 50 µm using a freezing slide microtome (Leica Biosystems Inc., Buffalo Grove, IL, USA). Sections were hand-mounted onto glass slides, dried overnight and stained for Nissl substance using thionin. The staining protocol included defatting (chloroform, 100% ethanol mixture, 1:1), pretreatment (acetic acid, acetone, 100% ethanol, double distilled water mixture, 1:1:1:1), staining (buffered thionin, 5%), differentiation (70% ethanol and 5–10 drops glacial acetic acid), dehydration (ethanol series: 70%, 95%, 100%), clearing (xylene) and coverslipping (Permount).

### Sampling and digitization

Guided by anatomical landmarks, we selected five sections per case spanning five different anterior to posterior levels (1, *genu*; 2, *pes*; 3, *full dentate gyrus*; 4, *x-region*; 5, *body*). These levels were further described with anatomical details identifying each of the five hippocampal sampling levels in previous studies.^13,29^ Thus, 35 sections (7 cases *×* 5 sections) were digitized at high resolution (×10 magnification) using a Keyence digital microscope (Keyence Corporation of America, Itasca, USA).

### Tissue parcellations

**Figure 1:**
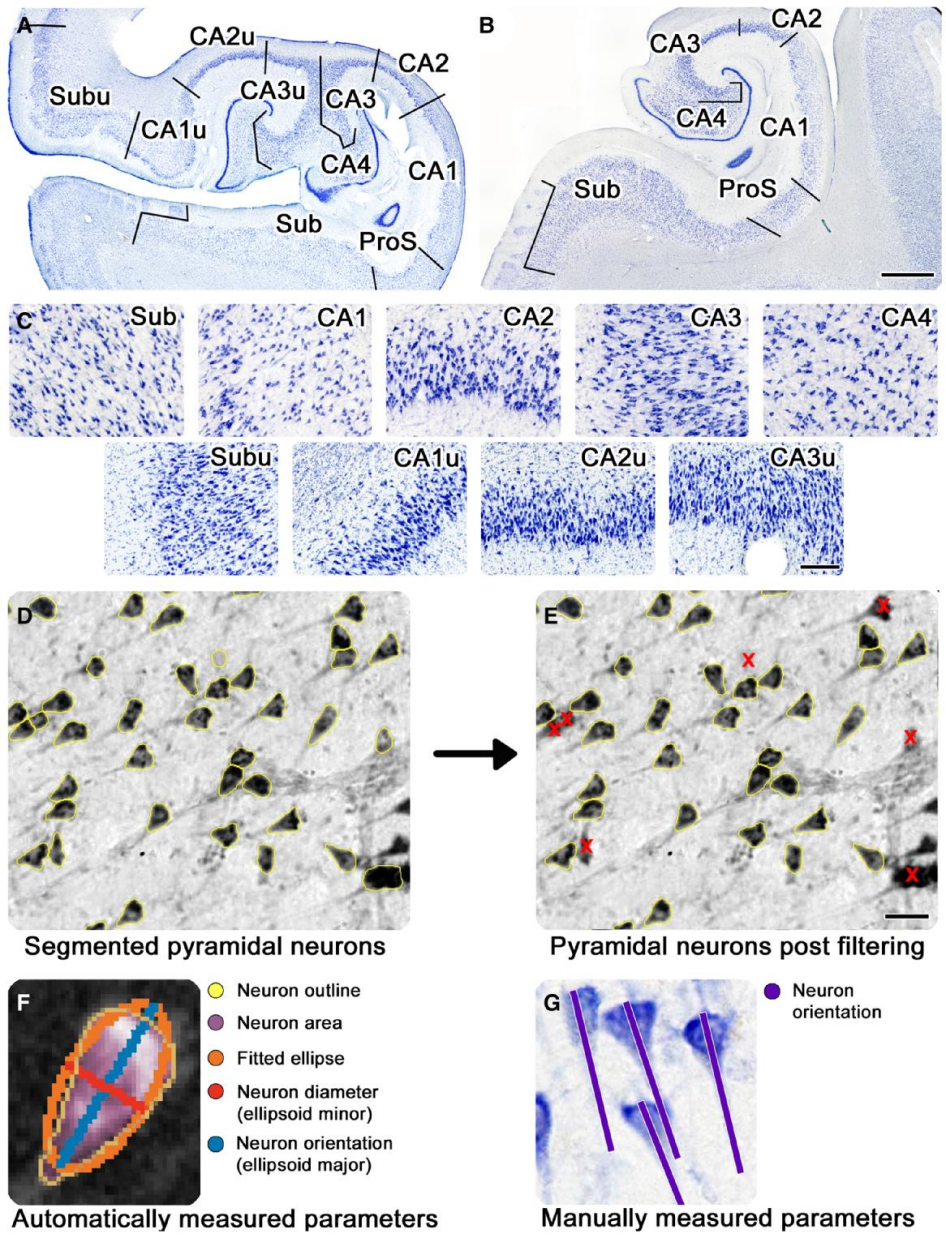
**Cytoarchitecture characteristics of hippocampal subregions in Nissl stain and overview for neuron parameters.** (**A**) Photomacrograph of the parcellated medial (uncal) and lateral subregions at the level of the hippocampal *x*-region. (**B**) Photomacrograph of parcellated lateral hippocampal subregions at the level of the hippocampal body. (**C**) Photomicrographs of hippocampal subregion cytoarchitecture characteristics. (**D**) Pyramidal neuron segmentations generated by Cellpose (unfiltered). (**E**) Filtered pyramidal neuron segmentations (X, excluded false-positive segmentation). (**F**) Ellipsoid-fitted hippocampal pyramidal neuron and extracted estimates. Colour code: yellow, neuron segmentation (neuron outline); purple, neuron area; orange, fitted ellipse; red, neuron diameter (ellipsoid minor); blue, neuron orientation (ellipsoid major). (**G**) Orientation lines of pyramidal neurons generated manually (purple line). Magnification bars in the photomacrograph = 2 mm, in the photomicrograph = 200 µm and in the vignettes illustrating the filtering procedures = 30 µm. CA1, cornu ammonis 1; CA2, cornu ammonis 2; CA3, cornu ammonis 3; CA4, cornu ammonis 4; CA1u, cornu ammonis 1 uncal; CA2u, cornu ammonis 2 uncal; CA3u, cornu ammonis 3 uncal; ProS, prosubiculum; Sub, subiculum.

Each sampled section was parcellated into subregions based on established architectural criteria.^10-13,16,30^ We defined the medial subregions as CA1u (CA1 uncal), CA2u (CA2 uncal), CA3u (CA3 uncal) and Subu (subiculum uncal) according to Ding *et al*.^11,13,31^ Williams *et al*.^13^ outline and detail the specific criteria-based parcellations. The data set used in this study contained multiple occurrences of each subregion totalling 168 individual partitions across cases, subregions and levels. While we differentiated prosubiculum from subiculum and CA1, it has not been included into the data set due to small neuron size.^32^ Figure 1A shows the cytoarchitectural characteristics of the medial and lateral subregions at the level of the hippocampal head, Fig. 1B displays the lateral subregions at the level of the hippocampal body, and Fig. 1C displays respective ×10 photomicrographs.

### Deep learning procedures

The pipeline consisted of the following: (i) preprocessing; (ii) automated segmentation of pyramidal neurons and elimination of false-positive segmentations; (iii) extraction of pyramidal neuron orientation estimates; and (iv) correction of pyramidal neuron orientations for the curvature of the hippocampus. Note that the collinearity measures represent a culmination that encapsulates the orientation of the individual neuron data. While (i), (ii) and (iii) were conducted using scripts generated for Fiji/ImageJ v1.53, the curvature correction and generation of collinearity measures was conducted using R-Studio v.1.4.1 (R-Studio Team, Boston, MA, packages ‘circular’ v.0.5–0. and ‘RANN’ v.2.6.1.). In-house developed scripts and plugins have been annotated and shared on GitHub to facilitate continued development and use (https://github.com/AugustinackLab/NeuronParameterEstimates).

#### Preprocessing

Each sampled hippocampal section was manually parcellated into subregions, digitized, transformed into 8-bit grey value, inverted and automatically adjusted for contrast and brightness to ensure optimal input images.^24,33^ Then, the pyramidal neuron layer was delineated and individual input partitions generated from the subregions. The preprocessing pipeline is illustrated in Supplementary Fig. 1.

#### Pyramidal neuron segmentation and exclusion of false-positive neurons

This study employed the pretrained deep neural network Cellpose (U-net architecture with residual blocks).^23^ Cellpose has previously been utilized for detailed histological analysis such as analysing myofibers,^34^ segmenting cell nuclei^35^ and quantifying hippocampal pyramidal neurons.^24^ All 168 hippocampal partitions were processed based on the optimal segmentation parameters for pyramidal neurons previously established in Oltmer *et al*.^24^  Supplementary Table 2 lists all Cellpose parameters utilized in our study. We identified and removed four potential instances of false-positive pyramidal neuron segmentations: (i) segmentations consisting of extracellular space were discarded by setting a threshold using the average grey value of extracellular space and neurons, thereby excluding the lighter extracellular space; (ii) glial cells (smaller than pyramidal neurons); (iii) neuron profiles (partial neurons, smaller than pyramidal neurons); and (iv) overlapping neurons (larger than one pyramidal neuron) were excluded based on deviation from the average segmentation size within a given partition. The lower size threshold was set at mean diameter—0.75 SD, while mean diameter + 1.75 SD and mean area + 1.75 SD were used as the upper threshold. Figure 1D shows a vignette of unfiltered pyramidal neuron segmentations, while Fig. 1E displays the same vignette after the exclusion of false-positive segmentations (yellow, neuron outlines; red X, excluded false positive). The structured piloting of optimal filtering thresholds based on the exclusion of false-positive segmentations without affecting accurately segmented pyramidal neurons was described in Oltmer *et al*.^24^  Supplementary Table 3 lists the various thresholds explored for optimal filtering performance. Individual segmentations were assigned a number and tallied into .tiff as well as .csv files for further analysis and quality control.

#### Pyramidal neuron orientation and size estimation

Each pyramidal neuron segmentation was ellipsoid fitted (Fig. 1F, orange ellipsoid).^36^ Given that pyramidal neurons have a triangular shape, the minor axis of the ellipse is width (Fig. 1F, red line), and the angle between the major axis (length) and the *x*-axis determines their orientation (Fig. 1F, blue line). Pyramidal neuron area was determined by the segmentation outline (Fig. 1F, purple area within the yellow outline).

#### Curvature correction of pyramidal neuron orientations and generation of collinearity measures

Hippocampal pyramidal neurons generally orient perpendicular to the pyramidal cell layer^12^ (stratum pyramidale of Lorente De Nó).^37^ Yet, the human brain, whether cortical or allocortical ribbon, has a complex morphometry. Consequently, the human hippocampus, and in turn the hippocampal pyramidal layer, exhibits varying levels of curvature due to its rolled up configuration.

**Figure 2:**
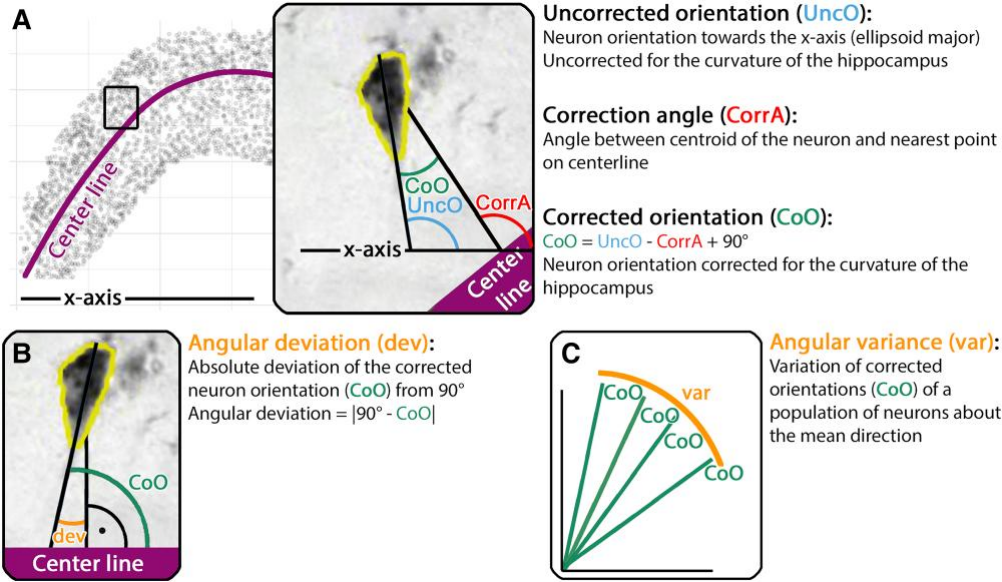
**Curvature correction of neuron orientations and resulting collinearity measures.** (**A**) Uncorrected pyramidal neuron orientation (UncO), correction angle (CorrA) and curvature corrected orientation (CoO). (**B**) The collinearity measure mean angular deviation (dev) described the mean deviation of the corrected orientations (CoOs) of the pyramidal neurons from 90° (perpendicular) towards the centreline of the pyramidal layer within a subregion. (**C**) As a second collinearity measure, angular variance (var) characterized the respective variance of pyramidal neuron CoOs above the mean. Colour code: center line, purple; uncorrected orientation (UncO), blue; correction angle (CorrA), red; corrected orientation (CoO), green; collinearity measures [variance (var) and mean angular deviation (dev)], orange.

Therefore, the orientation estimate of each individual neuron towards the *x*-axis (Figs. 1F and 2A), termed ‘uncorrected neuron orientation’ (UncO, blue line) needed to be corrected for the curved morphometry of the pyramidal layer to generate collinearity measures (Fig. 2). To this aim, the angle of the pyramidal neuron centroid (geometrical centre point) to the closest point on a manually drawn centreline of the pyramidal layer (Fig. 2, violet line) was calculated. This angle was termed ‘correction angle’ (Fig. 2, CorrA, red line). The UncO was then subtracted from the CorrA, resulting in the curvature corrected neuron orientation. The curvature corrected neuron orientation was termed ‘corrected neuron orientation’ (Fig. 2, CoO, green). Thus, CoO characterized the curvature corrected orientation of each individual pyramidal neuron towards the pyramidal layer based on the formula CoO = CorrA−UncO + 90° (Fig. 2A). For every subregion, two quantitative collinearity measures were computed from the individual CoOs: (i) mean angular deviation (Fig. 2B, dev, orange) indicated the deviation of the CoOs within a subregion from 90° as a reference angle of perfect orthogonality to the pyramidal layer and (ii) angular variance (Fig. 2C, var, orange) captured the variability of the respective CoOs above the mean angle^38^ (calculated using the var.circular function of the R ‘circular’ package). High levels of mean angular deviation or angular variance indicate a low level of neuronal collinearity within the respective subregion. Note that angular deviation allows us to run statistics, while angular variance gives an ideal overview of the spread of CoOs. To ensure quality control and validation, UncOs and CoOs of the pyramidal neurons within the individual partitions were graphed for comparison against manual orientation lines.

### Validation of automated pyramidal neuron collinearity measures

To validate our findings derived from automated pyramidal neuron collinearity measures, a subset of 10 sections was manually examined for pyramidal neuron collinearity (5 cases, each one section at the hippocampal body and head/dentate gyrus). First, Nissl sections were parcellated into subregions. Then, a systematic random sampling grid was applied (50 × 50 µm grid size) and 15% of grid squares sampled using a random number generator. For each neuron with a nucleolus located within a sampled grid, neuron orientation was then manually measured by drawing a line from the centre point between the basal dendrites towards its apical dendrite (Fig. 1G, violet line).^32^  Supplementary Fig. 2 illustrates the generation of manual pyramidal neuron orientation lines.

### Statistical analysis

Statistical analysis and data presentation were conducted using R-Studio v.1.4.1 (R-Studio Team, Boston, MA). Data presentation was conducted using Prism v.9.1 (GraphPad Software Inc., CA). We applied two linear mixed models (LME, R-package ‘lme4’ v.1.1–35.1) in combination with likelihood ratio tests to determine overall difference in angular deviation of the pyramidal neurons (Fig. 2B, dev) among the hippocampal subregions. Subregion as the variable of interest was applied as a fixed effect (Experiment 1: CA3, CA2, CA1, Sub, CA1u, CA2u, CA3u, Subu; Experiment 2: Proximal CA3, distal CA3, CA2), and case and slide (anterior–posterior level) were used as random effects to correct for pseudoreplication. Further information on the linear mixed models and likelihood ratio tests is shown in Supplementary Table 5. *Post hoc* testing for significant differences in angular deviations between subregions was conducted using a Tukey design, and results were corrected for multiple comparisons (R-package ‘multcomp’ v.1.4–25, single-step procedure). *P* < 0.05 was set as the level of significance.

To account for non-linearities in the variance and fulfil the assumptions of the linear effects models, the response variable (angular deviation) was transformed to resemble a normal distribution using a quantile normalization on the slide level (R-package ‘bestNormalize’ v.1.9.1)—mapping the quantiles of the distribution of angular deviations per slide to quantiles of normal distributions. A high effect indicates that the neurons of this subregion rank among the highest quantiles of angular deviations within each slide.

## Results

### Segmenting pyramidal neurons of the human hippocampal subregions using deep learning

We segmented 631 494 pyramidal neurons across 168 partitions (*n* = 168, 7 cases); 473 044 neuron segmentations remained post-filtering (70.00% ± 0.28; mean ± SEM), providing the basis to generate automated deep learning estimates. Supplementary Table 4 provides subregion-specific fractions of post-filtering segmentations.

### Manual orientation lines validate deep learning estimates of pyramidal neuron parameters

**Figure 3:**
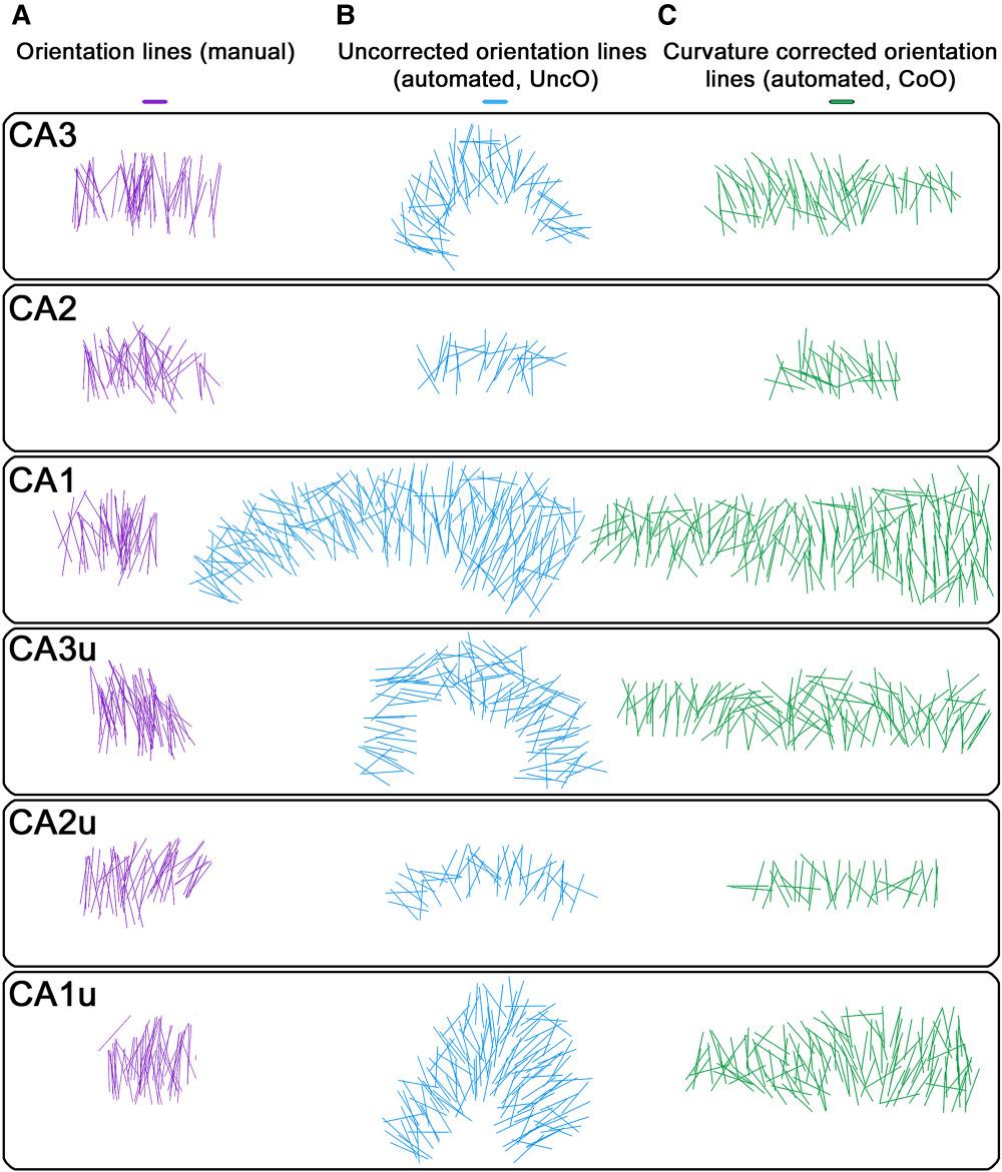
**Qualitative pyramidal neuron orientation line plots validate deep learning estimates.** (**A**) Manual pyramidal neuron orientation measures were drawn from the centre point between the basal dendrites to the apical dendrite of the neuron. Note that manual orientation lines only covered a smaller portion of the subfield, not its entire span. A systematic random sampling scheme from Stereoinvestigator (MicroBrightField Inc.) was used to select neurons and utilized inclusion and exclusion lines within the counting frame. Qualitative observations in manual orientation measures revealed that pyramidal neurons of CA3 were the most aligned, and CA1 and the uncal subregions showed the lowest collinearity. Per sampled partition, an approximate average of *n* = 50 pyramidal neurons have been manually assessed. (**B, C**) The same pattern of collinearity was observed in uncorrected orientation lines (automated) defined by the major axis of the fitted ellipse for each segmented neuron and curvature corrected orientation lines (automated). For **B** and **C**, one pyramidal neuron per grid square was randomly sampled (220 × 220 µm grid size) and the respective neuron orientation plotted (approximate average of *n* = 100 per partition). CA1, cornu ammonis 1; CA2, cornu ammonis 2; CA3, cornu ammonis 3; CA4, cornu ammonis 4; CA1u, cornu ammonis 1 uncal; CA2u, cornu ammonis 2 uncal; CA3u, cornu ammonis 3 uncal; dev, angular deviation; ProS, prosubiculum; Sub, subiculum; var, angular variance; CoO, corrected orientation; UncO, uncorrected orientation.

To confirm the accuracy of the automated neuron orientation estimates generated by the deep learning pipeline (UncO and CoO, Fig. 2), a comprehensive visual assessment was performed by comparing plots of UncO lines and curvature corrected neuron orientation lines with manual orientation line plots conducted for validation (five cases, each one section at the hippocampal body and head/dentate). Qualitative observations confirmed consistency in the pattern of manual and automated neuronal orientation lines (J.O., E.M.W. and J.C.A.). Figure 3 shows representative pyramidal neuron orientation line plots.

### Deep learning neuron collinearity measures help differentiate hippocampal subregions

**Figure 4:**
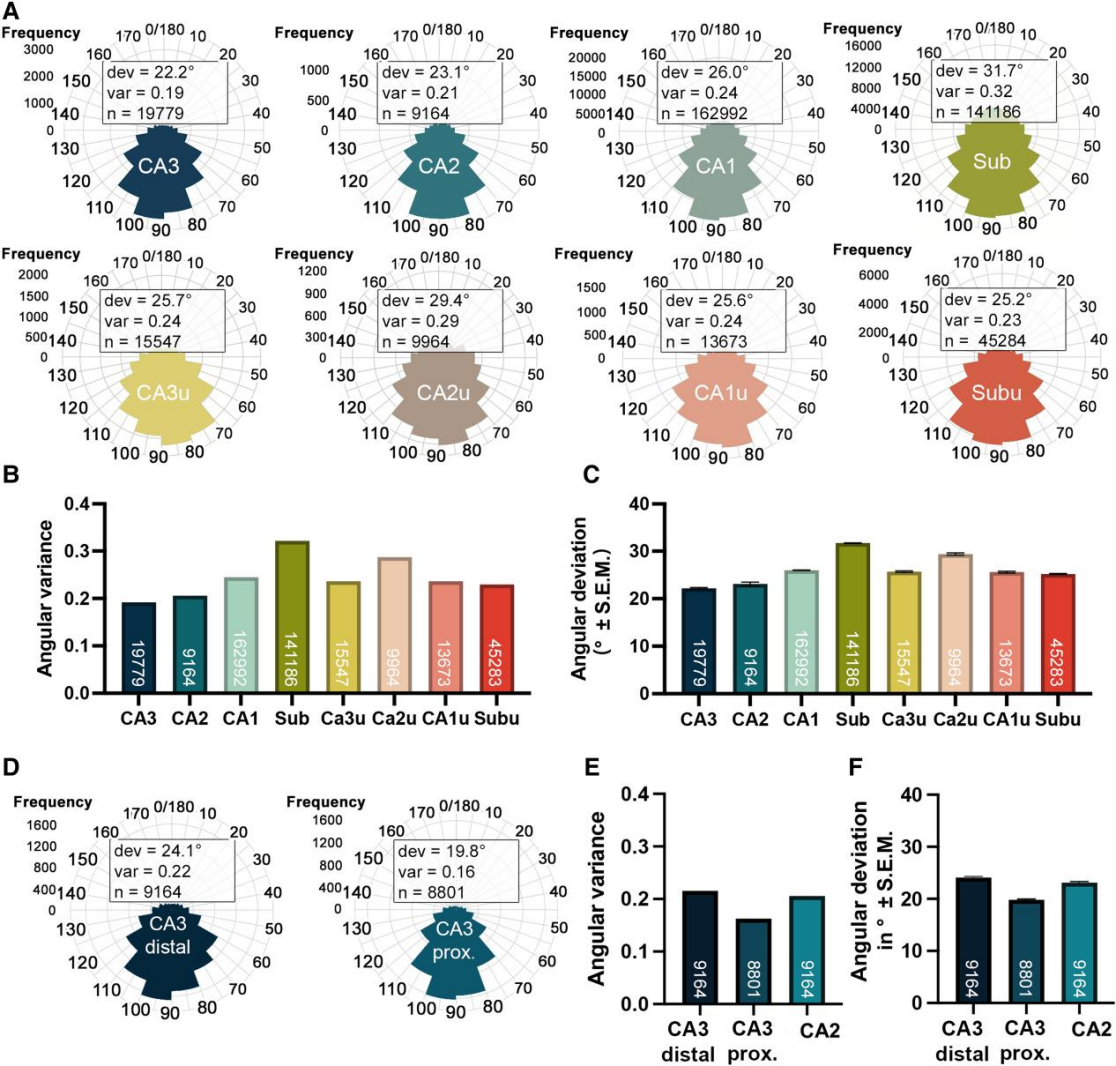
**Curvature corrected automated neuron collinearity estimates.** (**A**) Circular histogram (rose plots in R) displaying the frequency distribution, mean angular deviation (dev) and angular variance (var) of the curvature corrected neuron orientations (CoOs) within the collective hippocampal subregions. (**B, C**) Sub, CA2u and CA1 showed the least collinear neurons (largest angular variance and angular deviation), while CA3 was the most collinear (lowest angular variance and deviation). (**D**) Circular histogram displaying the frequency distribution, mean angular deviation (dev) and angular variance (var) of the curvature corrected neuron orientations (CoOs) within the collective distal CA3, proximal CA3 and CA2. (**E, F**) Angular variance and angular deviation of the curvature corrected neuron orientations was larger in distal CA3 (thus lower neuron collinearity), than in proximal CA3 and CA2 (greater neuron collinearity). The number of data points is denoted on the respective bars and rose plots. These data represent descriptive information. Table 2 and Supplementary Table 5 contain the statistical tests related to them. CA1, cornu ammonis 1; CA2, cornu ammonis 2; CA3, cornu ammonis 3; CA4, cornu ammonis 4; CA1u, cornu ammonis 1 uncal; CA2u, cornu ammonis 2 uncal; CA3u, cornu ammonis 3 uncal; CA3 prox, cornu ammonis 3 proximal; CA3 distal, cornu ammonis 3 distal; dev, angular deviation; ProS, prosubiculum; Sub, subiculum; var, angular variance.

Figure 2A shows the correction of neuron orientation estimates for the curved morphometry of the hippocampus (Fig. 2A). Figure 2B and C summarize the quantitative neuron collinearity measures within a hippocampal ribbon. Figure 3 shows the neuron orientation measures: manual, automated uncorrected (UncO) and automated curvature corrected (CoO). In the manual neuron orientation measures, automated uncorrected and automated curvature corrected orientation estimates, we observed a consistent pattern across methods. Generally, CA1 and the medial/uncal subregions had the lowest neuronal collinearity. In addition, we observed a gradient of neuronal collinearity within CA3, with distal CA3 (inner blade) showing lower collinearity than proximal CA3 (Fig. 3). Figure 4A shows circular histograms (rose plots in R) displaying the frequency distribution and the neuron collinearity measures: (i) mean angular deviation (dev) and (ii) angular variance (var) of the curvature corrected neuron orientations (CoOs) within the collective hippocampal subregions. Sub had the largest angular variance (indicating lowest neuron collinearity), followed by the medial/uncal subregions and CA1, CA2 and CA3 (Fig. 4A). The same pattern was observed for mean angular deviation (dev) (Fig. 4C). A linear mixed model approach in combination with a likelihood ratio test revealed a significant effect of subregion in angular deviation (likelihood ratio test: *P <* 0.001, *n* = 417 589 observations in 153 partitions, quantile normalized dev). Table 2 displays the respective direct comparisons, and Table 3 provides descriptive statistics of mean angular deviation (dev) within the collective hippocampal subregions. Additional details on the linear mixed models and likelihood ratio tests are displayed in Supplementary Table 5.

**Table 2:** Direct comparison of neuron collinearity between hippocampal subregions

Comparisons			Quantile normalized angular deviation			
	*n* partitions	*n* neurons	Estimate	Std. Error	*Z*-value	*P* adj.
CA1u versus CA1	**9 versus 35**	**13 673 versus 162 992**	**0**.**20**	**0**.**01**	**22**.**67**	**<0**.**001**
CA2 versus CA1	21 versus 35	9164 versus 162 992	0.02	0.01	2.11	0.372
CA2 versus CA1u	**21 versus 9**	**9164 versus 13 673**	**0**.**33**	**0**.**01**	**32**.**10**	**<0**.**001**
CA2u versus CA1	11 versus 35	9964 versus 162 992	−0.01	0.01	−1.78	0.595
CA2u versus CA1u	**11 versus 9**	**9964 versus 13 673**	**0**.**22**	**0**.**01**	**26**.**85**	**<0**.**001**
CA2u versus CA2	**11 versus 21**	**9964 versus 9164**	**0**.**22**	**0**.**00**	**60**.**25**	**<0**.**001**
CA3 versus CA1	**20 versus 35**	**19 779 versus 162 992**	**0**.**10**	**0**.**01**	**19**.**17**	**<0**.**001**
CA3 versus CA1u	**20 versus 9**	**19 779 versus 13 673**	**−0**.**18**	**0**.**01**	**−13**.**49**	**<0**.**001**
CA3 versus CA2	**20 versus 21**	**19 779 versus 9164**	**0**.**13**	**0**.**01**	**9**.**49**	**<0**.**001**
CA3 versus CA2u	**20 versus 11**	**19 779 versus 9964**	**−0**.**22**	**0**.**01**	**−19**.**45**	**<0**.**001**
CA3u versus CA1	10 versus 35	15 547 versus 162 992	0.02	0.01	1.71	0.643
CA3u versus CA1u	10 versus 9	15 547 versus 13 673	0.02	0.01	2.31	0.255
CA3u versus CA2	**10 versus 21**	**15 547 versus 9164**	**−0**.**10**	**0**.**01**	**−10**.**45**	**<0**.**001**
CA3u versus CA2u	**10 versus 11**	**15 547 versus 9964**	**0**.**31**	**0**.**01**	**21**.**32**	**<0**.**001**
CA3u versus CA3	10 versus 20	15 547 versus 19 779	−0.04	0.01	−2.85	0.071
Sub versus CA1	**35 versus 35**	**141 186 versus 162 992**	**0**.**20**	**0**.**01**	**15**.**41**	**<0**.**001**
Sub versus CA1u	**35 versus 9**	**141 186 versus 13 673**	**0**.**20**	**0**.**01**	**18**.**87**	**<0**.**001**
Sub versus CA2	**35 versus 21**	**141 186 versus 9164**	**0**.**08**	**0**.**01**	**7**.**00**	**<0**.**001**
Sub versus CA2u	**35 versus 11**	**141 186 versus 9964**	**−0**.**34**	**0**.**01**	**−28**.**03**	**<0**.**001**
Sub versus CA3	**35 versus 20**	**141 186 versus 19 779**	**−0**.**11**	**0**.**01**	**−8**.**26**	**<0**.**001**
Sub versus CA3u	**35 versus 10**	**141 186 versus 15 547**	**−0**.**10**	**0**.**01**	**−10**.**12**	**<0**.**001**
Subu versus CA1	**12 versus 35**	**45 284 versus 162 992**	**−0**.**23**	**0**.**01**	**−20**.**61**	**<0**.**001**
Subu versus CA1u	**12 versus 9**	**45 284 versus 13 673**	**0**.**24**	**0**.**01**	**22**.**27**	**<0**.**001**
Subu versus CA2	**12 versus 21**	**45 284 versus 9164**	**0**.**24**	**0**.**01**	**31**.**36**	**<0**.**001**
Subu versus CA2u	**12 versus 11**	**45 284 versus 9964**	**0**.**12**	**0**.**01**	**13**.**59**	**<0**.**001**
Subu versus CA3	12 versus 20	45 284 versus 19 779	0.00	0.01	0.07	>0.999
Subu versus CA3u	**12 versus 10**	**45 284 versus 15 547**	**−0**.**12**	**0**.**01**	**−13**.**19**	**<0**.**001**
Subu versus Sub	**12 versus 35**	**45 284 versus 141 186**	**−0**.**12**	**0**.**01**	**−22**.**82**	**<0**.**001**
CA3 prox. versus CA2	**20 versus 21**	**8801 versus 9164**	**−0**.**16**	**0**.**02**	**−10**.**86**	**<0**.**001**
CA3 dist. versus CA2	**20 versus 21**	**9164 versus 9164**	**0**.**04**	**0**.**01**	**2**.**80**	**0**.**014**
CA3 dist. versus CA3 prox.	**21 versus 21**	**9164 versus 8801**	**0**.**20**	**0**.**01**	**14**.**25**	**<0**.**001**

To account for non-linearities in the variance and align with the assumptions of the linear effects model, we conducted quantile normalization at the slide level. A high effect implies that neurons in this region rank among the highest quantiles of angular deviations within the slide. *P*-values were adjusted for multiple comparisons. Bold lettering highlights significant differences. CA4 was excluded due to the location in the hilus and is not organized into a layer. Prosubiculum was not included due to small neuron size.

*n* neurons, total number of neurons; Std. Error, standard error; *Z*-value, *Z* test statistic; *P* adj., adjusted *P*-value.

**Table 3:** Descriptive statistics of neuron collinearity among the hippocampal subregions

Pyramidal layer neuron variable of interest, unit	Subregion	*n* partitions	*n* neurons	25% perc.	Median	75% perc.	Mean	SD	SEM	Lower 95% CI	Upper 95% CI
Angular deviation, °	CA1	35	162 992	20.52	23.28	26.55	24.79	5.86	0.99	22.78	26.80
	CA1u	9	13 673	22.90	27.55	28.38	25.45	4.81	1.60	21.75	29.15
	CA2	21	9164	19.51	21.35	26.52	22.79	4.85	1.06	20.58	25.00
	CA2u	11	9964	23.23	28.99	35.66	28.61	7.24	2.18	23.74	33.48
	CA3	20	19 779	20.26	22.30	23.51	21.73	3.59	0.80	20.05	23.41
	CA3u	10	15 547	21.39	24.08	26.86	24.45	4.24	1.34	21.42	27.48
	Sub	35	141 186	25.47	28.46	38.39	30.97	7.83	1.32	28.28	33.66
	Subu	12	45 284	22.96	25.74	27.43	25.31	3.07	0.89	23.36	27.26
	CA3 proximal	20	8801	17.10	19.04	20.59	19.09	3.59	0.80	17.41	20.77
	CA3 distal	20	9164	21.99	24.09	25.72	23.61	4.42	0.99	21.54	25.68

Subregion, number of partitions (*n* partitions), total number of neurons (*n* neurons), 25% percentile, median, 75% percentile, mean, standard deviation (SD), standard error of the mean (SEM), lower 95% confidence interval (CI) and upper 95% confidence interval of automated angular deviation estimates.

We specifically investigated differences in neuron collinearity between CA3 and CA2, given that CA2 and CA3 neurons show similar features (both stain darkly with large size). CA3 displayed a lower mean angular deviation and angular variance than CA2 (CA3: dev = 22.2°, var = 0.19; CA2: dev = 23.1°, var = 0.21) (Table 3 and Fig. 4). The difference in quantile normalized angular deviation was significant (*P* < 0.001, *Z* = 9.49) (Table 2). To further account for the gradient in neuronal collinearity within CA3, we characterized the lower collinearity in the distal CA3 compared to the proximal CA3 and CA2. Figure 4D displays circular histograms (rose plots in R) illustrating the frequency distribution of the curvature corrected neuron orientations (CoOs) and the respective collinearity measures mean angular deviation (dev) and angular variance (var) within proximal CA3 and distal CA3. We observed lower angular variance and mean angular deviation in proximal CA3 compared to CA2 and distal CA3 (distal CA3: dev = 24.1°, var = 0.22; proximal CA3: dev = 19.8°, var = 0.16; CA2: dev = 23.1°, var = 0.21) (Fig. 4E and F). The linear mixed model approach in combination with a likelihood ratio test showed a significant effect of subregion in angular deviation (likelihood ratio test: *P <* 0.001, *n* = 28 943 observations in 61 partitions, quantile normalized dev), and we observed significantly lower values in proximal CA3 in comparison to distal CA3 (*P* < 0.001, *Z* = 14.25) or CA2 (*P* < 0.001, Z = −10.86). Distal CA3 and CA2 were significantly different as well (*P* = 0.014, *Z* = 2.80) (Tables 2 and 3).

## Discussion

This study presents a high-throughput deep learning pipeline that distinguishes individual subregions within the human hippocampus based on the collinearity of pyramidal neurons. We utilized the pretrained Cellpose algorithm for cellular segmentation^23^ in combination with vetted input parameters and established filtering methods^24^ to segment and measure 479 873 pyramidal neurons in 168 hippocampal partitions (sample set). The hippocampus has a complex morphometry, and its pyramidal layer displays substantial curvature. Thus, we report ‘curvature corrected’ collinearity measures generated automatically but validated to manual measures. Our automated approach revealed unique differences between the individual hippocampal subregions, establishing pyramidal neuron collinearity as a novel quantitative parameter for hippocampal subregion segmentation.

Deep learning has provided new opportunities for automation in histopathology. It has been used for cancer classification,^39,40^ the identification of histopathological markers,^41,42^ myofiber segmentation,^34^ and hippocampal pyramidal neuron quantification.^24^ While human evaluation is fundamental to the advancement of science, it is labour-intensive and may be influenced by various human factors such as subjectivity, differences in training, recognition biases and fatigue.^43,44^ Automated deep learning approaches possess the potential to drastically reduce manual labour. However, validating these methods is crucial. In a previous study, we demonstrated that pyramidal neuron segmentations generated by Cellpose did not differ significantly in dice scores from manual segmentations. Moreover, we demonstrated that automated neuron number estimates strongly correlated with manual stereology total neuron counts.^24^ In the current study, a consistent pattern of collinearity was observed across all methods when applying qualitative but criteria-based comparisons of neuron orientation line plots from (i) manual neuron orientation, (ii) automated UncO and (iii) curvature corrected neuron orientation (J.O., E.M.W. and J.C.A.) (Fig. 3). In conclusion, our findings indicate that the presented deep learning approach generated quantitative data of pyramidal neuron collinearity which was on par with manual measures.

Neuron collinearity, as an architectonic feature, has not been evaluated in many disease conditions. Collinearity may be a prospective feature for analysing architecture in post-mortem samples from psychiatric conditions. Deviations in pyramidal neuron collinearity within the hippocampal subregions have been reported in schizophrenia.^20,21^ Williams *et al*.^13^ observed collinearity differences among the hippocampal subregions using a criteria-based architectural approach. In addition, previous methods for the measurement of pyramidal neuron collinearity relied on relatively small neuron numbers,^20-22^ or predefined ranges of pixel colour and intensity (grey value thresholding),^21^ highlighting the need for a more quantitative approach. These initial studies introduced the observed abnormality of neuron collinearity in schizophrenia—future studies may apply the automated approach detailed here to find additional correlations with mental health and disease.

This study tackled the curvature of the hippocampus, creating meaningful orientation measures. It is important to highlight that, to the best of our knowledge, no previous studies have applied curvature correction to neuronal orientation estimates to produce curvature corrected subregional collinearity measures. This curvature correction process is essential for comparisons between individual cases and among hippocampal subregions. This study reports a quantitative metric for curvature corrected neuron collinearity within the human hippocampal subregions. Our findings demonstrate significant differences in collinearity among subregions (Fig. 4). Specifically, pyramidal neurons of Sub exhibited the largest angular deviation (31.7°) and angular variance (0.32), while CA3 pyramidal neurons showed the most collinear alignment (angular deviation: 22.2°; angular variance: 0.19). Among the hippocampal subregions, CA3 pyramidal neurons appear extremely organized and aligned, which was confirmed with our neuron orientation plots of the individual hippocampal subregions (Fig. 3).

Our results have implications for hippocampal parcellation. So far, many studies group CA2 and CA3 together, possibly due to a lack of quantitative differentiation parameters.^45-49^ Our study demonstrated significant differences in pyramidal neuron collinearity between CA2 and CA3 (comparing quantile normalized angular deviation in whole CA3 as well as the proximal and distal regions of CA3 to CA2). The proximal region of CA3 exhibited less angular deviation than both CA2 and the distal region of CA3. Our automated quantitative results contribute to data that CA2 and CA3 show different cytoarchitecture organization^13,50^ and may aid in future hippocampal parcellation in big data sets.

This study acknowledges several limitations. Previous work demonstrated that Cellpose combined with specific input and filtering parameters segments pyramidal neurons with high accuracy compared to manual raters.^24,34^ Yet, false-positive segmentations may occur (extracellular space, glial cells, partial neurons and combined pyramidal neurons). To address this, we excluded false-positive segmentations based on filtering methods described in Oltmer *et al*.^24^ This study assesses the hippocampal cytoarchitecture in the coronal plane, which is in plane with the organization of pyramidal neurons.^12-14,51^ However, a small fraction of neurons on the fringe of the pyramidal layer may be oriented differently and were excluded from the analyses. Variability in histology staining quality may impact automated deep learning image analysis among cases; histochemistry staining occurs because of natural affinity, which sometimes varies among samples. This is a common occurrence when working with the human brain, attention for this is imperative for good staining as input for computational analyses. A human rater may be more resilient to changes in stain quality and adapt accordingly. The automated pipeline employed in this study utilizes a deep neural network to segment and measure pyramidal neurons, taking into account differences in shapes and patterns in image data, which creates a regimented approach against these potential biases.

Deep learning methods have the potential to reduce labour, facilitate multicentric cohorts, reduce interrater variability and might aid in the future evaluation of large-scale data sets.^34,41-44^ Deep learning is expected to facilitate and shape future medical research and practice.^52,53^ This study implemented and validated a novel deep learning approach for the characterization and differentiation of the human hippocampal subregions based on differences in pyramidal neuron collinearity. Further experiments may utilize provided scripts (GitHub) and these published parameters, thereby supporting forthcoming comparisons within and between individuals in large data sets. Future studies might also explore potential application in neuropathology sections. Finally, the automated assessment of neuron collinearity could aid future studies in identifying architectonic changes in mental illness and neurodegenerative diseases—providing insight on the cellular level.

## Supplementary Material

fcae296_Supplementary_Data

## Data Availability

The data sets generated during and/or analysed during the current study are available from the corresponding author upon reasonable request. The scripts and plugins generated for this study are available on GitHub (https://github.com/AugustinackLab/NeuronParameterEstimates).
