# Supplementary material for: Neuron collinearity differentiates human hippocampal subregions: a validated deep learning approach
Source: Brain Commun. 2024 Sep 3;6(5):fcae296. doi: 10.1093/braincomms/fcae296 (PMC11389610; doi:10.1093/braincomms/fcae296)
Supplement: fcae296_Supplementary_Data [file fcae296_supplementary_data.pdf]

Neuron Collinearity Differentiates Human Hippocampal Subregions: a Validated Deep Learning Approach

Oltmer, Jan[1,2,3], Williams, Emily M[1], Groha, Stefan[2,4], Rosenblum, Emma W[1], Roy, Jessica[1], Llamas-Rodriguez, Josue[1], Perosa, Valentina[5], Champion, Samantha N[6], Frosch, Matthew P[6], Augustinack, Jean C[1,2]

Author affiliations:

1 Athinoula A. Martinos Center, Massachusetts General Hospital, Department of Radiology, Charlestown, MA, USA

2 Harvard Medical School, Boston, MA, USA

3 Department of Digital Health & Innovation, Vivantes Netzwerk für Gesundheit GmbH, Berlin, Germany

4 Division of Population Sciences, Department of Medical Oncology, Dana-Farber Cancer Institute, Boston, MA, USA

5 Department of Neurology, Massachusetts General Hospital, Harvard Medical School, J. Philip Kistler Stroke Research Center, Cambridge Str. 175, Suite 300, Boston, MA, 02114, USA

6 C.S. Kubik Laboratory for Neuropathology, Massachusetts General Hospital, Boston, MA, USA

Correspondence to:

Jean C Augustinack

Full address: Department of Radiology, Athinoula A. Martinos Center for Biomedical Imaging, Massachusetts General Hospital, Building 149 –13th St. Room 2301, Charlestown, MA, 02129, USA

E-mail: jaugustinack@mgh.harvard.edu

Reagent	***Source***	***Identifier***
Acetic Acid, Glacial, Certified ACS	Fisher Scientific	A38-500
Acetone	Fisher Scientific	HC3001GAL
Chloroform, Certified ACS	Fisher Scientific	C298-1
Dimethyl Sulfoxide, Certified ACS	Fisher Scientific	D128-500
100% Denatured Ethyl Alcohol	Fisher Scientific	HC8001GAL
95% Denatured Ethyl Alcohol	Fisher Scientific	HC11001GL
Glycerol	SIGMA	G9012-1L
L-Lysine Hydrochloride	Fisher Scientific	BP386-100
Sodium-meta-periodate 99%	Fisher Scientific	AC19838-1000
Sodium phosphate dibasic dihydrate	SIGMA	71505-250G
Sodium phosphate monobasic heptahydrate	SIGMA	S2429-250G
Paraformaldehyde 96%	Fisher Scientific	AC41678-5000
Permount	Fisher Scientific	SP15-500
Thionin Acetate, pure, high purity biological stain	Fisher Scientific	AC22984-0050
Sodium Acetate Trihydrate, ACS Reagent Grade	Fisher Scientific	RDCS0250500
Xylenes, Histological Grade	Fisher Scientific	X3P-1GAL
AT8 monoclonal antibody	Fisher Scientific	MN1020
Peroxidase AffiniPure Goat Anti-Mouse IgG (H+L)	Jackson Immuno Research	115-035-003
3'3-Diaminobenzidine	Vector Laboratories	SK-4100
Vectasian Elite ABC kit (standard)	Vector Laboratories	PK-6100

Supplementary Table 1. Reagents and sourcing: Reagents used, source and catalog identifier.

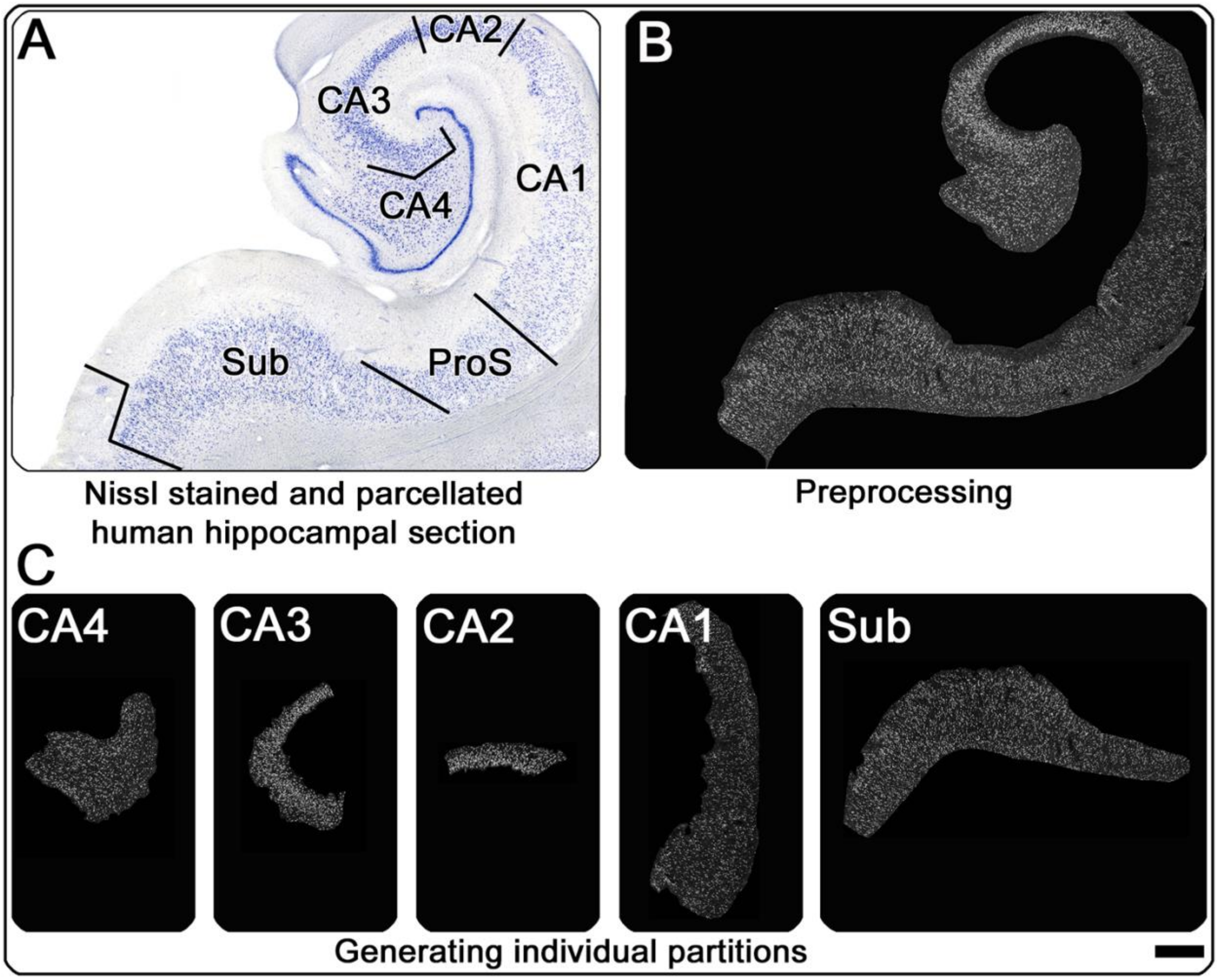

Supplementary Figure 1. Preprocessing pipeline for optimal Cellpose input images: A) Parcellated Nissl stained hippocampal section (coronal plane, 50µm thick). B) Hippocampal pyramidal neuron layer, transformed into an 8-bit gray-value image and inverted. C) Individual partitions (various subfields) created from the pyramidal layer. Magnification bar = 1mm

Input parameter	***Value***
pixel diameter	24
model_type	cyto
pretrained_model	true
diameter	24
flow_threshold	0.4
mask_threshold	0.0
resample	false
use_gpu	true
save_png	true

Supplementary Table 2. Input parameters used for the neuronal segmentation using CellPose.

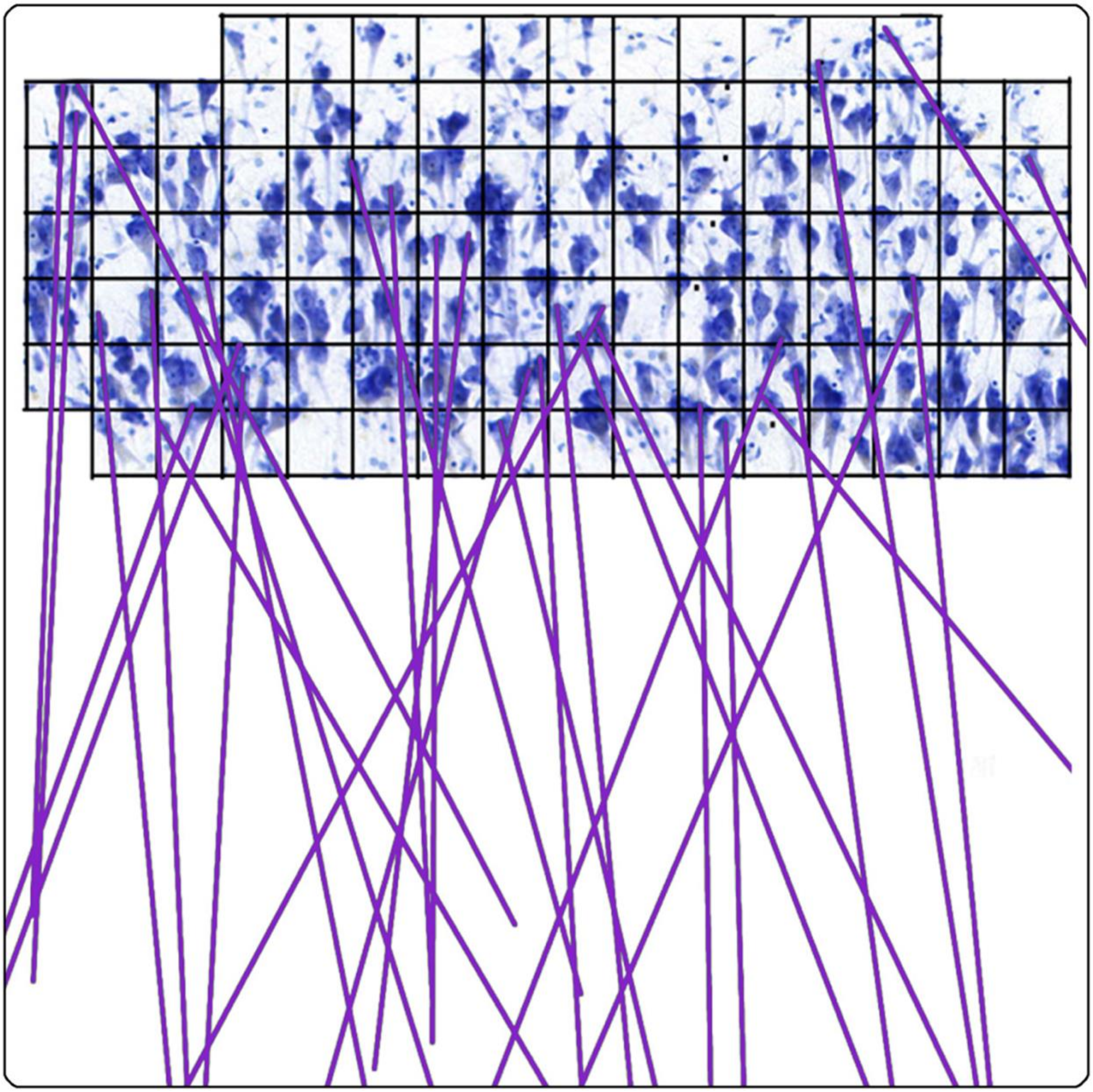

Supplementary Figure 2. Manual neuron orientation measurement: Manual measurements of pyramidal neuron orientation were collected in five cases (5 cases x 2 levels). Per partition, a systematic random sampling grid was applied (50µm x 50 µm grid size), and approximately 15% of grid squares were randomly sampled. Pyramidal neuron orientations were then manually drawn from the midpoint of the base of the pyramidal neuron through the apical dendrite. Black boxes = sampling grid, purple lines = manual pyramidal neuron orientation measure.

Lower diameter thresholds	*Upper diameter thresholds*	*Lower area thresholds*	*Upper area thresholds*
- 0.5 SD	1 SD	0.75 SD	1 SD
- 0.75 SD *	1.75 SD *	1 SD	1.75 SD *
- 1 SD	2 SD	2 SD	2 SD
- 2 SD	2.5 SD		2.5 SD
	3 SD		3 SD

Supplementary Table 3. Various filtering parameters investigated for the automated exclusion of false positive segmentations: Lower diameter, upper diameter, lower area, and upper area thresholds were assessed per partition based on the deviation of the segmentation diameter or area from the mean. Filtering results were then ranked for exclusion of false positive segmentations while leaving pyramidal neurons untouched. Note, -0.75 SD diameter (lower threshold), in combination with +1.75 SD diameter and +1.75 SD area (upper thresholds) were selected and applied as best performing filtering parameters (marked with an asterisk).

Pyramidal layer neuron variable of interest, Unit	*Sub-region*	*n partitions*	*25% Perc.*	*Median*	*75% Perc.*	*Mean*	*SD*	*S.E.M.*	*Lower 95% CI*	*Lower 95% CI*
Filtered neuron count estimates, %	CA1	35	67.89	71.06	73.93	70.11	5.26	0.89	68.31	71.92
	CA1u	9	68.81	70.25	71.86	70.03	2.20	0.73	68.34	71.73
	CA2	21	68.87	69.95	72.52	70.40	2.18	0.48	69.40	71.39
	CA2u	11	69.28	70.66	72.27	70.63	1.41	0.43	69.69	71.58
	CA3	20	70.85	71.69	72.25	71.56	1.56	0.35	70.83	72.29
	CA3u	10	70.34	70.83	71.56	70.67	1.66	0.52	69.49	71.86
	CA4	16	62.83	71.56	73.40	68.88	5.67	1.42	65.86	71.90
	Sub	35	66.59	69.54	70.74	68.55	3.15	0.53	67.47	69.64
	Subu	12	67.04	70.69	71.58	69.86	2.59	0.75	68.22	71.51

Supplementary Table 4. Percentage of hippocampal pyramidal layer neurons post filtering: Table 2. Descriptive statistics of the hippocampal subregions: Subregion, number of partitions (n partitions), 25% percentile, median, 75% percentile, mean, SD, S.E.M., lower 95% confidence interval, and upper 95% confidence interval of automated pyramidal neuron count estimates post filtering.

Effect of subregion on angular deviation within the hippocampal subregions (CA1 | CA1u | CA2 | CA2u | CA3 | CA3u | Sub | Subu)

model: Angular-deviation ~ subregion + (1 | case) + (1 | slide)

null-model: Angular-deviation ~ (1 | case) + (1 | slide)

	npar	AIC	BIC	logLik	deviance	Chisq	Df	P-value
**null-model**	4	1184694	1184738	-592343	1184686			
**model**	11	1179267	1179388	-589623	1179245	5440.4	7	<0.001

Effect of subregion on angular deviation within the hippocampal subregions of CA3 and CA2 (CA3 proximal | CA3 distal | CA2)

model: Angular-deviation ~ subregion + (1 | case) + (1 | slide)

null-model: Angular-deviation ~ (1 | case) + (1 | slide)

	npar	AIC	BIC	logLik	deviance	Chisq	Df	P-value
**null-model**	4	82117	82150	-41055	82109			
**model**	6	81905	81954	-40946	81893	216.5	2	<0.001

Supplementary Table 5 - Linear mixed models and likelihood-ratio-tests for the individual analysis. To address non-linear variance and fulfill the assumptions of linear effects models, angular deviation was normalized to approximate a normal distribution (quantile normalization at the slide level. Mixed-effects models were constructed with the effect of interest (model) and without (null-model). Multlple measures per subject were accounted for using a random-intercept model. Model fit was affirmed by examining the distribution of residuals via QQ-plots and histograms. Comparisons were conducted using Likelihood-Ratio-Tests. Abbreviations: npar = number of parameters, AIC = Akaike Information Criterion, BIC = Bayesian Information Criterion. loglik = Log-Likelihood, Chisq = $\chi 2$ test statistic, Df = degrees of freedom.
